# A Preliminary Study on Metabolome Profiles of Buffalo Milk and Corresponding Mozzarella Cheese: Safeguarding the Authenticity and Traceability of Protected Status Buffalo Dairy Products

**DOI:** 10.3390/molecules25020304

**Published:** 2020-01-12

**Authors:** Angela Salzano, Gelsomina Manganiello, Gianluca Neglia, Francesco Vinale, Donato De Nicola, Michael D’Occhio, Giuseppe Campanile

**Affiliations:** 1 Dipartimento di Medicina Veterinaria e Produzioni Animali, University of Naples “Federico II”, 80137 Naples, Italy; angela.salzano@unina.it (A.S.); frvinale@unina.it (F.V.); denicolavet@gmail.com (D.D.N.); giucampa@unina.it (G.C.); 2 Dipartimento di Agraria, University of Naples “Federico II”, 80055 Portici (NA), Italy; gelsomina.manganiello@hotmail.it; 3 Istituto per la Protezione Sostenibile delle Piante, National Research Council, 80100 Naples, Italy; 4 Faculty of Science, The University of Sydney, Sydney, NSW 2006, Australia; michael.docchio@sydney.edu.au

**Keywords:** metabolome, GC-MS, buffalo, milk, mozzarella, authenticity

## Abstract

The aim of this study is to combine advanced GC-MS and metabolite identification in a robust and repeatable technology platform to characterize the metabolome of buffalo milk and mozzarella cheese. The study utilized eleven dairies located in a protected designation of origin (PDO) region and nine dairies located in non-PDO region in Italy. Samples of raw milk (100 mL) and mozzarella cheese (100 g) were obtained from each dairy. A total of 185 metabolites were consistently detected in both milk and mozzarella cheese. The PLS-DA score plots clearly differentiated PDO and non-PDO milk and mozzarella samples. For milk samples, it was possible to divide metabolites into two classes according to region: those with lower concentrations in PDO samples (galactopyranoside, hydroxybuthyric acid, allose, citric acid) and those with lower concentrations in non-PDO samples (talopyranose, pantothenic acid, mannobiose, etc.,). The same was observed for mozzarella samples with the proportion of some metabolites (talopyranose, 2, 3-dihydroxypropyl icosanoate, etc.,) higher in PDO samples while others (tagatose, lactic acid dimer, ribitol, etc.,) higher in non-PDO samples. The findings establish the utility of GC-MS together with mass spectral libraries as a powerful technology platform to determine the authenticity, and create market protection, for “Mozzarella di Bufala Campana.”

## 1. Introduction

The authenticity, integrity, and traceability of food products is very important for market protection in global food systems [1,2,3,4]. This applies particularly to food that has a market niche and for which consumers pay a high price. The premium price of niche food makes it susceptible to substitution with falsely-labelled, non-authentic food [5]. Examples are substitution in olive oil [6,7] and dairy products [8,9,10]. Italian mozzarella cheese is made from the milk of Italian Mediterranean water buffalo (*Bubalus bubalis*) and is recognized globally for its exceptional eating qualities. The water buffalo undergo intense selection for efficiency of production and product quality, and today has an important commercial and cultural niche in several regions of Italy. Genuine Italian mozzarella is known as “Mozzarella di Bufala Campana.” It has European Union protected designation of origin (PDO) and protected geographical indication (PGI) status. This identifies genuine mozzarella with four regions in Italy (Apulia, Campania, Lazio, Molise) [11,12]. Attempts are regularly made to place non-authentic mozzarella in premium markets as a substitute for buffalo mozzarella cheese [13,14,15]. Consumers can be denied a genuine product and confidence in certified farmers is threatened. The potential for product substitution has led to interest in the development of screening technology that ensures the authenticity of buffalo mozzarella and traceability to geographical origin [14,16,17].

The characterization of the metabolome of food is a promising approach for establishing authenticity [18]. The metabolome signature of biological material can be obtained using gas chromatography/mass spectrometry (GC-MS) [19,20], NMR spectroscopy [21,22], and high-resolution magic angle spinning (HRMAS) NMR spectroscopy [23]. Some initial screening of buffalo mozzarella cheese has used NMR [16], HRMAS NMR [16], and GC-MS/LC-MS [14,15,17]. To date, individual studies have looked at the metabolome of either buffalo milk or mozzarella. Substitution can occur at different stages of the supply chain and it is important that authenticity can be established from primary product (milk) through to secondary product (mozzarella). There are also limited studies in dairy on the impacts of breeding, diet, and geographical location on product quality [24]. The present study sought to compare, for the first time, the metabolomes of both unprocessed milk and corresponding mozzarella cheese for buffalo in PDO and non-PDO regions in Italy. Differences in the milk and/or mozzarella metabolomes between buffalo in PDO and non-PDO regions have potential practical application in safeguarding the authenticity and traceability of protected status buffalo dairy products.

## 2. Results

**Figure 1:**
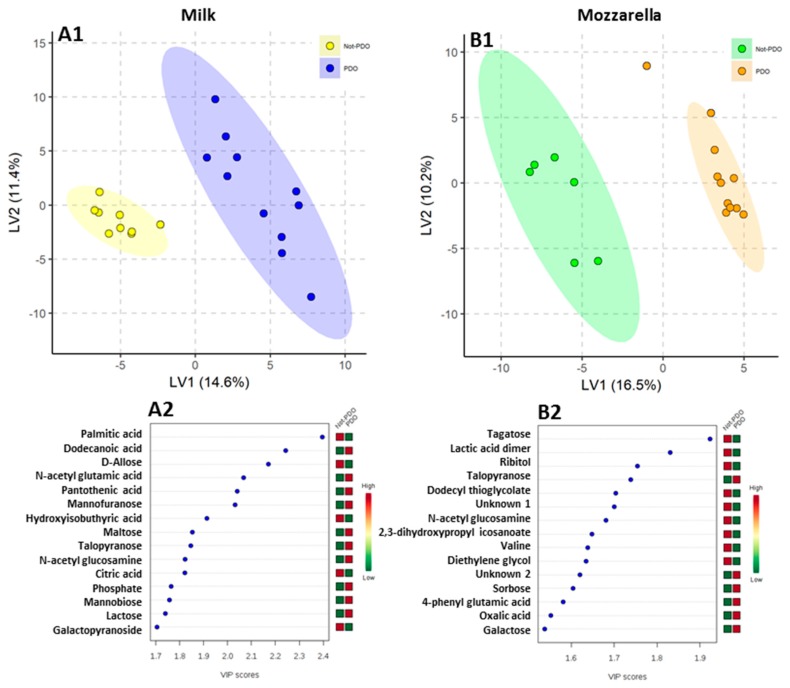
Partial least square discriminant analysis (PLS-DA) models to discriminate PDO and non-PDO buffalo milk (**A1**) and mozzarella (**B1**) samples. The explained variance of each component is shown in parentheses on the corresponding axis. (**A2**) and (**B2**) panels show the 15 top-scoring variable importance in projection (VIP) metabolites (VIP-score ≥ 1.5) for milk (A2) and mozzarella (B2) samples. The colored boxes on the right indicate the relative amount of the corresponding metabolite in each group under study.

No differences were observed in the quality of milk and mozzarella cheese from PDO and non-PDO regions (Table 1 and Table 2). A total of 185 metabolites were detected consistently. In particular, 113 compounds were detected in milk, 102 in mozzarella cheese, and 30 compounds out of 185 were found in both matrices. The PLS-DA score plots clearly differentiated PDO and non-PDO milk and mozzarella samples (Figure 1(A1,B1)). The 15 highest scoring variable importance in projection VIP variables (VIP score > 1.5) identified by PLS-DA are shown in Figure 1(A2,B2).

**Figure 2:**
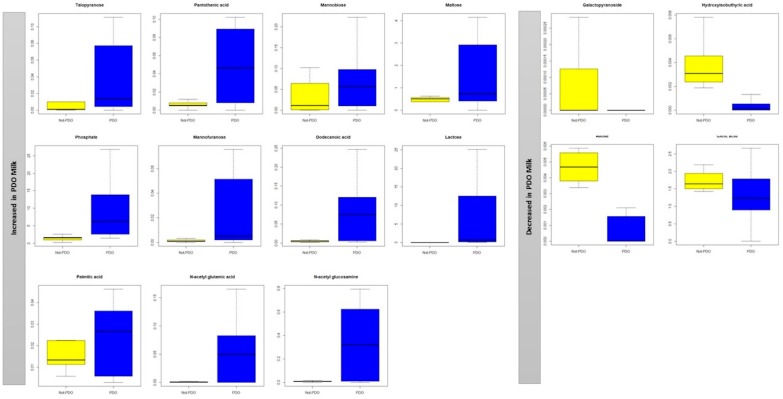
Box and Whisker plot of the VIP metabolites in buffalo milk samples. Boxes represent non-PDO (yellow) and PDO (blue) milk samples. The vertical axis reports the log of the gas chromatography mass spectrometry values of the normalized area of each metabolite.

Differences in metabolite concentrations between PDO and non-PDO raw milk samples allowed the metabolites to be separated into two classes: those with lower (*p* < 0.05) concentrations in PDO milk (galactopyranoside, hydroxybuthyric acid, allose, citric acid) and those with higher (*p* < 0.05) concentrations in PDO milk (talopyranose, pantothenic acid, mannobiose, maltose, phosphate, mannofuranose, dodecanoic acid, lactose, palmitic acid, n-acetyl glutamic acid, *n*-acetyl glucosamine) (Figure 2).

**Figure 3:**
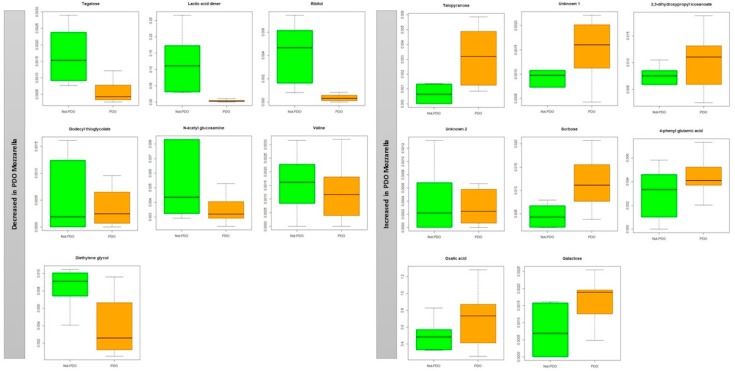
Box and Whisker plot of the VIP metabolites in buffalo mozzarella samples. Boxes represent non-PDO (green) and PDO (orange) mozzarella samples. The vertical axis reports the log of the gas chromatography mass spectrometry values of the normalized area of each metabolite.

Metabolites could also be separated into two classes for mozzarella cheese samples: those with higher (*p* < 0.05) concentrations in PDO mozzarella (talopyranose, 2, 3-dihydroxypropyl icosanoate, sorbose, 4-pnehyl glutamic acid, oxalic acid, galactose) and those with higher (*p* < 0.05) concentrations in non-PDO mozzarella (tagatose, lactic acid dimer, ribitol, dodecyl thioglycolate, *n*-acetyl glucosamine, valine, diethylene glycol) (Figure 3).

## 3. Discussion

The present study sought to compare the metabolomes of unprocessed milk and corresponding mozzarella cheese for buffalo in PDO and non-PDO regions in Italy. It was found that the milk metabolome differed between buffalo in PDO and non-PDO regions. The mozzarella metabolome also differed between buffalo in PDO and non-PDO regions. This is the first time that some metabolites have been detected in both the metabolome of unprocessed milk and corresponding mozzarella cheese in buffalo. It is also the first time that differences have been found between buffalo in PDO and non-PDO regions in both milk and mozzarella metabolomes. The number of individual farms from PDO (n = 11) and non-PDO (n = 9) regions might be considered a relatively small sample size, although it was comparable to numbers in previous reports that looked at the metabolome [24,25,26]. Notwithstanding the sample size, the combination of GC-MS and mass spectral libraries (NIST library) proved to be a robust technology platform for determining the metabolomes of buffalo milk and mozzarella. This platform, together with a rigorous analysis of the data, has provided a sound foundation to inform further studies. In particular, a number of notable metabolites identified in unprocessed milk (n = 15) and mozzarella cheese (n = 13) could be used to validate the utility of the metabolome for safeguarding the protected status buffalo dairy products. Candidate metabolites that differentiated PDO from non-PDO milk included several carbohydrates (d-allose, mannofuranose, maltose, and talopyranose). Candidate metabolites that differentiated both milk and mozzarella between PDO and non-PDO origin were talopyranose and *N*-acetyl glucosamine. Talopyranose is the pyranose form of talose and an epimer of glucose. Talose was already reported to be a “differential” marker between bovine milk and goat milk [27]. *N*-acetylglucosamine is a derivative amide of the monosaccharide glucose and a secondary amide between glucosamine and acetic acid. It is significant in several biological systems and a major component of the cell walls of most fungi [28]. Talopyranose is of particular interest as amounts were substantially higher in both milk and mozzarella of PDO origin. A number of saccharides (tagatose, talopyranose, sorbose, galactose) differentiated mozzarella of DPO and non-DPO origin. Two metabolites in mozzarella cheese had markedly different concentrations between PDO and non-PDO samples. These metabolites could not be identified from public mass spectral libraries. However, they require further study as they may emerge as highly valuable in establishing the authenticity and integrity of protected status buffalo mozzarella cheese.

Irrespective of PDO or non-PDO origin, the milk aqueous fraction was rich in short-chain saturated carboxylic acids (carbonic acid, acetic acid, propanoic acid, butanoic acid, octanoic acid, decanoic acid) and long-chain saturated carboxylic acids (lauric acid, palmitic acid, stearic acid). Free amino acids in milk included serine, threonine, and valine. Compared with milk, mozzarella cheese contained lower amounts of some short-chain saturated carboxylic acids (acetic, propanoic, nonanoic acid) and only two long-chain saturated carboxylic acids were found in mozzarella (palmitic acid, stearic acid). Mozzarella was, however, richer in free amino acids (serine, leucine, isoleucine, alanine, proline, valine, norvaline). Some of the metabolites found in mozzarella samples in the present study (lactic acid dimer, valine, oxalic acid, and galactose) were also identified in a study that looked at the metabolomic and microbiological differences of Italian mozzarella cheese produced with buffalo or cow milk [29].

Processing alters milk constituents and their concentrations. For example, milk monosaccharide levels change in response to heat treatment [30] and storage [31]. The composition of the starter, which can be influenced by the environment, management and cheese-making technologies, also affects cheese characteristics by altering the milk quality. [32,33,34]. In PDO mozzarella, there may have been a synergistic effect between different lactic acid bacteria and yeast species, which ferment residual galactose and lactose and thereby increase lactic acid production. The processed cheese industry uses citrate or phosphate salts to sequester Ca^2+^ from residual colloidal calcium phosphate. This solubilizes caseins which can then emulsify fat globules. The acidity of whey at drainage, and the rate of acid development, are important parameters that determine the mineral content, acidity, and quality of cheese [35]. Milk of PDO origin had higher phosphate content and lower citric acid compared to non-PDO milk. Differences in milk salt composition most likely contributed to the differences in metabolites between PDO and non-PDO mozzarella cheese. Differences in packaging could also modify the metabolomic profile of mozzarella cheese. The higher diethylene glycol concentration in samples of non-PDO mozzarella compared to PDO was attributed to packaging [36].

Climatic conditions influence the rate of uptake of metabolites by plants [37,38]. Also, lower temperatures and frequent rainfall can affect the drying process of forages; essentially, reducing sugars, mineral salts, and soluble nitrogenous substances, while increasing fermentation [39]. Rainfall is usually less frequent in the PDO regions in the present study compared with the non-PDO regions, and the higher concentrations of some sugars (maltose, lactose, mannobiose, talopyranose) in milk of PDO origin may have been due to differences in climatic conditions. The climate and soils of a region directly and indirectly impact the biochemical and biophysical properties of food products. For example, the isotopic and elemental composition of milk is closely related to geographical origin and this carries over to the properties of cheese [39]. To have PDO certification, at least 70% of the dry matter of fodder, or 40% of the dry matter of the ration, must come from the PDO region. The use of local fodder helps to maintain the strict relationship between product and region for protected status of buffalo mozzarella cheese. There are close relationships between the environment, rumen microbiome, and animal metabolome. While the major families of microbes in the rumen are broadly similar across diverse landscapes [40], the relative populations of different microbes change according to local conditions of climatic, soil, feed, and management [40,41,42]. The amount of crude protein, neutral-detergent fiber, and acid detergent lignin are higher in cultivars from the PDO region [43]. It can be assumed that this would have influenced the rumen microbial populations and ruminal metabolome, which could have contributed, at least in part, to differences in the milk and mozzarella metabolomes between PDO and non-PDO regions.

In conclusion, a robust GC-MS and mass spectral library technology platform was used to identify for the first time the metabolome of unprocessed milk and corresponding mozzarella cheese in buffalo. Differences in both the milk and mozzarella metabolomes between buffaloes in PDO and non-PDO regions were also shown for the first time. A number of candidate metabolites in milk and mozzarella were identified that will be important in validation studies that aim to develop practical protocols to distinguish between PDO and non-PDO buffalo milk and mozzarella. Talopyranose was a particularly notable candidate metabolite as it differed substantially between PDO and non-PDO buffalo, for both milk and mozzarella. The development of quality assurance and certification protocols for milk and cheese will help to ensure the authenticity and traceability of primary (milk) and secondary (mozzarella) protected status buffalo dairy products. This is necessary to ensure that the investment in breeding, feeding, and management of buffalo in PDO regions is safeguarded.

## 4. Materials and Methods

### 4.1. Sample Collection

The study utilized 20 commercial buffalo dairies. Eleven dairies were located in a protected designation of origin (PDO, Campania, Italy) region and nine dairies were in non-PDO regions in Italy (). All dairies had a processing facility that produced mozzarella cheese exclusively from their own milk. Milk and mozzarella cheese quality was assessed the day of the sampling and the average results from PDO and non-PDO areas are reported in Table 1 and Table 2. Pooled samples of raw milk (100 mL) and mozzarella cheese (100 g) were obtained from each dairy. The samples underwent similar processing and were obtained approximately 2 h after preparation. Mozzarella samples were immersed in mozzarella whey and stored at 25 °C until analysis.

### 4.2. Metabolomics Analysis

#### 4.2.1. Metabolite Extraction and Derivatization

Metabolite extraction, purification, and derivatization were carried out using the MetaboPrep GC kit according to the manufacturer’s instructions (Theoreo srl, Montecorvino Pugliano [SA], Italy).

From each mozzarella sample 10 ± 1 mg was transferred to an Eppendorf microcentrifuge tube containing the extraction solution. The samples were then centrifuged at 800× *g* for 30 min, before putting the samples in an ultrasonic bath at 30 °C for 30 min. The samples were then centrifuged for 5 min at 10,000× *g* at 4 °C. From the supernatant, 200 μL was removed and transferred to an Eppendorf microcentrifuge tube containing a purification mixture, and then vortexed at 800× *g* for 30 sec. The sample was again centrifuged at 10,000× *g* (at 4 °C). Finally, 175 μL supernatant was transferred into a 2-mL glass autosampler vial and freeze-dried overnight.

To facilitate derivatization, “50 µL of pyridine/methoxamine (1/1 *v*:*v*) were added to the sample and centrifuged at 800× *g* (25 °C) for 90 min. Then 25 µL of the second derivatization mixture containing *N*,*O*-*bis*(trimethylsilyl)trifluoroacetamide (BSTFA) and trimethylchlorosilane (TMCS) were added and vortexed at 800× *g* (25 °C) for 90 min. The solution was centrifuged for 5 min at 10,000 rpm× *g* (4 °C) before injecting into the GC-MS.”

#### 4.2.2. GC-MS Analysis

Samples (2 µL) of the derivatized solution were injected into the GC-MS system (GC-2010 Plus gas chromatograph coupled to a 2010 Plus single quadrupole mass spectrometer; Shimadzu Corp., Kyoto, Japan). Chromatographic separation was achieved with a 30 m 0.25 mm CP-Sil 8 CB fused silica capillary GC column with 1.00 µm film thickness (Agilent, J&W Scientific, Folsom, CA, USA), with helium as carrier gas. The initial oven temperature of 100 °C was maintained for 1 min and then raised by 6 °C/min to 320 °C with a further 2 min of holding time. The gas flow was set to obtain a constant linear velocity of 39 cm/s and the split flow was set at 1:5. The mass spectrometer was operated with electron impact ionization (70 eV) in full scan mode in the interval of 35–600 *m*/*z* with a scan velocity of 3333 AMU/sec and a solvent cut time of 4.5 min. The complete GC program duration was 40 min.

### 4.3. Metabolites Identification

Metabolite identification was performed according to Troisi et al. [44]. Briefly, untargeted metabolites were identified by comparing the mass spectrum of each peak with the NIST library collection (NIST, Gaithersburg, MD, USA). The linear index difference max tolerance was set to 10, while the minimum matching spectra library search was set to 85% (level 2 identification according to Metabolomics Standards Initiative [MSI]) [45]. Fifteen samples out of the over 200 signals per sample (7.5%) produced by gas chromatographic–mass spectrometry were not investigated further because they were not consistently found in other sets of samples (either too low in concentration or of poor spectral quality to be confirmed as metabolites).

### 4.4. Statistical Analyses

Data regarding milk and mozzarella cheese quality are expressed as mean ± SE. Differences were assessed by Student’s t-test, and *p* < 0.05 value was considered significant. The chromatographic data were tabulated with one sample per row and one variable (metabolite) per column. Data pre-treatment consisted of normalizing each metabolite peak area to that of the internal standard (2-iso-propyl malic acid) followed by generalized log transformation and data scaling by autoscaling (mean-centered and divided by standard deviation of each variable). Statistical analysis of data from three biological replicates for each farm was performed by ANOVA Bonferroni correction (*p*-value < 0.05) by applying GraphPad PRISM software. Only metabolites significantly different between the farms were considered for further analyses. Principal component analyses (PCA) and heatmap representations were conducted by the online tool ClustVis (http://biit.cs.ut.ee/clustvis/). Unit variance scaling was applied to rows (metal/metabolite values in each farm) and single value decomposition (SVD) with imputation was used to calculate principal components. The heatmaps were generated clustering columns (Farms) by correlation distance and McQuitty linkage. Samples were classified considering geographical area of origin (North/South) and also the presence or absence of PDO trademark (Yes = Y; No = N).

Moreover, partial least square discriminant analysis (PLS-DA) [46] was performed using the statistical software package R (Foundation for Statistical Computing, Vienna, Austria). Class separation was achieved by PLS-DA, which is a supervised method that uses multivariate regression techniques to extract, via linear combinations of original variables (X), the information that can predict class membership (Y). PLS regression was performed using the plsr function included in the R pls package [47]. Classification and cross-validation were performed using the corresponding wrapper function included in the caret package [48]. A permutation test was performed to assess the significance of class discrimination. In each permutation, a PLS-DA model was built between the data (X) and the permuted class labels (Y) using the optimal number of components determined by cross validation for the model based on the original class assignment. Variable importance in projection (VIP) scores were calculated for each metabolite. The VIP score is a weighted sum of squares of the PLS loadings, taking into account the amount of explained Y-variation in each dimension.

## Figures and Tables

**Table 1:** Average composition of milk samples from different farms located in protected destination of origin (PDO) and non-PDO regions in Italy. Data are expressed as means ± ES.

BUFFALO MILK					
Nutrition Facts for 100 g of Product (Reg. UE 1169/2011)	NON-PDO REGION (n = 9)		PDO REGION (n = 11)		*p* Value
	U.M	VALUE	U.M	VALUE	
Energy net value	KJ/100 g	473 ± 8.4	KJ/100 g	479 ± 7.9	0.82
	Kcal/100 g	112 ± 3.5	Kcal/100 g	116 ± 3.7	0.77
Total protein	g/100 g	4.5 ± 0.7	g/100 g	4.6 ± 0.6	0.85
Total fat	g/100 g	8.4 ± 0.9	g/100 g	8.6 ± 0.8	0.69
Saturated fat	g/100 g	4.9 ± 0.5	g/100 g	4.9 ± 0.5	0.91
Total carbohydrates	g/100 g	5.2 ± 0.4	g/100 g	5.2 ± 0.5	0.82
Sugars	g/100 g	0.8 ± 0.1	g/100 g	0.9 ± 0.1	0.86
Salt	g/100 g	Nd	g/100 g	Nd	
Ashes	g/100 g	0.8 ± 0.0	g/100 g	0.8 ± 0.1	0.90

U.M. Unit measure.

**Table 2:** Average mozzarella cheese composition of samples from different farms located in protected designation of origin (PDO) and non-PDO regions in Italy. Data are expressed as means ± ES.

BUFFALO MOZZARELLA CHEESE					
Nutrition Facts for 100 g of Product (Reg. UE 1169/2011)	NON-PDO REGION (n = 9)		PDO REGION (n = 11)		*p* Value
	U.M.	VALUE	U.M.	VALUE	
Energy net value	KJ/100 g	1129.9 ± 9.4	KJ/100 g	1131.1 ± 8.2	0.72
	Kcal/100 g	268.6 ± 4.0	Kcal/100 g	269.2 ± 3.2	0.84
Total protein	g/100 g	13.4 ± 1.0	g/100 g	13.5 ± 0.9	0.86
Total fat	g/100 g	23.3 ± 2.6	g/100 g	23.5 ± 2.9	0.73
Saturated fat	g/100 g	14.6 ± 0.4	g/100 g	14.6 ± 0.6	0.79
Total carbohydrates	g/100 g	0.8 ± 0.0	g/100 g	0.8 ± 0.0	0.91
Sugars	g/100 g	0.8 ± 0.1	g/100 g	0.8 ± 0.0	0.88
Salt	g/100 g	0.9 ± 0.1	g/100 g	0.9 ± 0.1	0.90
Ashes	g/100 g	1.4 ± 0.2	g/100 g	1.4 ± 0.2	0.93

U.M. Unit measure.

## References

[1] Cubero-Leon E., Peñalver R., Maquet A. (2014). Review on metabolomics for food authentication. Food Res. Int..

[2] Danezis G.P., Tsagkaris A.S., Brusic V., Georgiou C.A. (2016). Food authentication: State of the art and prospects. Curr. Opin. Food Sci..

[3] Danezis G.P., Tsagkaris A.S., Camin F., Brusic V., Georgiou C.A. (2016). Food authentication: Techniques, trends & emerging approaches. TrAC Trends Anal. Chem..

[4] Pustjens A., Muilwijk M., Weesepoel Y., van Ruth S. (2016). Advances in Authenticity Testing of Geographical Origin of Food Products. Advances in Food Authenticity Testing.

[5] Álvarez B., Pascual-Alonso M., Rusu A., Bogason S. (2013). A review on existing databases relevant for food fraud and authenticity. Arch. Zootec..

[6] Tsopelas F., Konstantopoulos D., Kakoulidou A.T. (2018). Voltammetric fingerprinting of oils and its combination with chemometrics for the detection of extra virgin olive oil adulteration. Anal. Chim. Acta.

[7] Merás I.D., Manzano J.D., Rodríguez D.A., de la Peña A.M. (2018). Detection and quantification of extra virgin olive oil adulteration by means of autofluorescence excitation-emission profiles combined with multi-way classification. Talanta.

[8] Agrimonti C., Pirondini A., Marmiroli M., Marmiroli N. (2015). A quadruplex PCR (qxPCR) assay for adulteration in dairy products. Food Chem..

[9] Azad T., Ahmed S. (2016). Common milk adulteration and their detection techniques. Int. J. Food Contam..

[10] Gonçalves J., Pereira F., Amorim A., van Asch B. (2012). New method for the simultaneous identification of cow, sheep, goat, and water buffalo in dairy products by analysis of short species-specific mitochondrial DNA targets. J. Agric. Food Chem..

[11] Bonetti E. (2004). The effectiveness of meta-brands in the typical product industry: Mozzarella cheese. Br. Food J..

[12] Hajdukiewicz A. (2014). European Union agri-food quality schemes for the protection and promotion of geographical indications and traditional specialities: An economic perspective. Folia Hortic..

[13] Pignata M.C., Ferrão S.P.B., Oliveira C.P., Faleiro A.S., Bonomo R.C., Silva W.S., Rodrigues L.B., de Albuquerque Fernandes S.A. (2015). Mechanical Parameters of the Mozzarella from Buffalo with Inclusion Levels of The Cow’s Milk: Preliminary Study at the Lab Scale. J. Bioanal. Biomed..

[14] Caira S., Pinto G., Nicolai M.A., Chianese L., Addeo F. (2016). Simultaneously tracing the geographical origin and presence of bovine milk in Italian water buffalo Mozzarella cheese using MALDI-TOF data of casein signature peptides. Anal. Bioanal. Chem..

[15] Russo R., Severino V., Mendez A., Lliberia J., Parente A., Chambery A. (2012). Detection of buffalo mozzarella adulteration by an ultra-high-performance liquid chromatography tandem mass spectrometry methodology. J. Mass Spectrom..

[16] Mazzei P., Piccolo A. (2012). 1H HRMAS-NMR metabolomic to assess quality and traceability of mozzarella cheese from Campania buffalo milk. Food Chem..

[17] Sassi M., Arena S., Scaloni A. (2015). MALDI-TOF-MS platform for integrated proteomic and peptidomic profiling of milk samples allows rapid detection of food adulterations. J. Agric. Food Chem..

[18] Cevallos-Cevallos J.M., Reyes-De-Corcuera J.I., Etxeberria E., Danyluk M.D., Rodrick G.E. (2009). Metabolomic analysis in food science: A review. Trends Food Sci. Technol..

[19] Gianferri R., Maioli M., Delfini M., Brosio E. (2007). A low-resolution and high-resolution nuclear magnetic resonance integrated approach to investigate the physical structure and metabolic profile of Mozzarella di Bufala Campana cheese. Int. Dairy J..

[20] He Q., Kong X., Wu G., Ren P., Tang H., Hao F., Huang R., Li T., Tan B., Li P. (2009). Metabolomic analysis of the response of growing pigs to dietary L-arginine supplementation. Amino Acids.

[21] Nicholson J., Lindon J. (2008). Metabonomics. Nature.

[22] Sacco D., Brescia M., Sgaramella A., Casiello G., Buccolieri A., Ogrinc N., Sacco A. (2009). Discrimination between Southern Italy and foreign milk samples using spectroscopic and analytical data. Food Chem..

[23] Shintu L., Ziarelli F., Caldarelli S. (2004). Is high-resolution magic angle spinning NMR a practical speciation tool for cheese samples? Parmigiano Reggiano as a case study. Magn. Reson. Chem..

[24] Sun H.-Z., Wang D.-M., Wang B., Wang J.-K., Liu H.-Y., Guan L.L., Liu J.-X. (2015). Metabolomics of four biofluids from dairy cows: Potential biomarkers for milk production and quality. J. Proteome Res..

[25] Caboni P., Murgia A., Porcu A., Manis C., Ibba I., Contu M., Scano P. (2019). A metabolomics comparison between sheep’s and goat’s milk. Food Res Int..

[26] Caboni P., Murgia A., Porcu A., Demuru M., Pulina G., Nudda A. (2016). Gas chromatography-mass spectrometry metabolomics of goat milk with different polymorphism at the αS1-casein genotype locus. J. Anim. Sci..

[27] Pisano M.B., Scano P., Murgia A., Cosentino S., Caboni P. (2016). Metabolomics and microbiological profile of Italian mozzarella cheese produced with buffalo and cow milk. Food Chem..

[28] Scano P., Murgia A., Pirisi F.M., Caboni P. (2014). A gas chromatography-mass spectrometry-based metabolomic approach for the characterization of goat milk compared with cow milk. J Dairy Sci..

[29] Chen J.K., Shen C.R., Liu C.L. (2010). N-acetylglucosamine: Production and applications. Mar. Drugs.

[30] Mendoza M.R., Olano A., Villamiel M. (2005). Chemical indicators of heat treatment in fortified and special milks. J. Agric. Food Chem..

[31] Troyano E., Villamiel M., Olano A., Sanz J., Martínez-Castro I. (1996). Monosaccharides andmyo-inositol in commercial milks. J. Agric. Food Chem..

[32] Coppola S., Parente E., Dumontet S., La Peccerella A. (1988). The microflora of natural whey cultures utilized as starters in the manufacture of Mozzarella cheese from water–buffalo milk. Le Lait.

[33] Coppola S., Blaiotta G., Ercolini D., Moschetti G. (2001). Molecular evaluation of microbial diversity occurring in different types of Mozzarella cheese. J. Appl. Microbiol..

[34] Bonizzi I., Feligini M., Aleandri R., Enne G. (2006). Genetic traceability of geographical origin of typical Italian water buffalo mozzarella cheese: A preliminary approach. J. App. Microbiol..

[35] Lucey J.A., Johnson M.E., Horne D.S. (2003). Invited review: Perspectives on the basis of the rheology and texture properties of cheese. J Dairy Sci..

[36] MEGlobal Fast Facts Diethylene Glycol. http://www.meglobal.biz/media/MEGlobal_FastFacts_DEG.pdf.

[37] Kabata-Pendias A., Pendias H. (2001). Trace Elements in Soils and Plant.

[38] Rotz C.A., Muck R.E., Fahey G.C., Collins M., Mertens D.R., Moser L.E. (1994). Changes in forage quality during harvest and storage. Forage Quality, Evaluation, and Utilization.

[39] Bontempo L., Paolini M., Franceschi P., Ziller L., García-González D.L., Camin F. (2019). Characterisation and attempted differentiation of European and extra-European olive oils using stable isotope ratio analysis. Food Chem..

[40] Henderson G., Cox F., Ganesh S., Jonker A., Young W., Abecia L., Angarita E., Aravena P., Arenas G.N., Ariza C. (2015). Rumen microbial community composition varies with diet and host, but a core microbiome is found across a wide geographical range. Sci. Rep..

[41] Jami E., White B.A., Mizrahi I. (2014). Potential role of the bovine rumen microbiome in modulating milk composition and feed efficiency. PLoS ONE.

[42] Lima F.S., Oikonomou G., Lima S.F., Bicalho M.L., Ganda E.K., de Oliveira Filho J.C., Lorenzo G., Trojacanec P., Bicalho R.C. (2015). Prepartum and postpartum rumen fluid microbiomes: Characterization and correlation with production traits in dairy cows. Appl. Environ. Microbiol..

[43] Berardo N. (1997). Prediction of the chemical composition of white clover by near-infrared reflectance spectroscopy. Grass Forage Sci..

[44] Troisi J., Sarno L., Martinelli P., Di Carlo C., Landolfi A., Scala G., Rinaldi M., D’Alessandro P., Ciccone C., Guida M. (2017). A metabolomics-based approach for non-invasive diagnosis of chromosomal anomalies. Metabolomics.

[45] Sumner L.W., Amberg A., Barrett D., Beale M.H., Beger R., Daykin C.A., Fan T.W.-M., Fiehn O., Goodacre R., Griffin J.L. (2007). Proposed minimum reporting standards for chemical analysis Chemical Analysis Working Group (CAWG) Metabolomics Standards Initiative (MSI). Metabolomics Off. J. Metabolomic Soc..

[46] Wold S., Sjöström M., Eriksson L. (2001). PLS-regression: A basic tool of chemometrics. PLS Methods.

[47] Mevik B.H., Wehrens R. (2007). The pls Package: Principal Component and Partial Least Squares Regression in R. J. Stat. Softw..

[48] Kuhn M. (2008). Building Predictive Models in R Using the caret Package. J. Stat. Softw..

